# Muscle-Strengthening Activities and Sociodemographic Correlates among Adults: Findings from Samples in Mainland China

**DOI:** 10.3390/ijerph17072266

**Published:** 2020-03-27

**Authors:** Youliang Lin, Jin Yan

**Affiliations:** 1 Department of Physical Education, Wuhan University of Technology, Wuhan 430070, China; 2 Faculty of Education and Arts, University of Newcastle, Callaghan, NSW 2308, Australia; jin.yan@uon.edu.au

**Keywords:** physical activity epidemiology, muscle activity, factors, Chinese adults, sex difference

## Abstract

A growing body of research has investigated the level of participation in muscle-strengthening (MS) activities and their correlates among Western populations; however, scarce attention has been paid to this issue among Chinese adults. This study aimed to describe the level of MS activities and to explore the relationships between sociodemographic correlates and level of MS activities in a large sample of Chinese adults. For this study, 3073 adults were recruited from 13 cities in Hubei Province. A self-reported questionnaire was utilized to collect data on MS activities and sociodemographic information among participants in this study. According to World Health Organization physical activity guidelines, MS activities should be undertaken at least two days per week. Multivariate logistic regression was used to explore the sociodemographic correlates of MS activities. The statistical significance level was set up as *p* < 0.05. The prevalence of MS activities among participants was 28.5%. MS activities among the total samples were associated with sex (adjusted odds ratios (aORs) for male = 1.98, 95% confidence intervals (95% CI): 1.67–2.34) and family composition (aOR for multiple children = 1.35, 95%CI: 1.12–1.64). Among males, normal weight status (aOR = 1.39, 95%CI: 1.08–1.78) and multiple children (aOR = 1.58, 95% CI: 1.21–2.05) were associated with MS activities. There was no association of sociodemographic factors with MS activities among females. Our results suggest that approximately 70% of adults in Hubei Province do not engage in recommended MS activities. These activities were associated with sex and family composition, which differed between sexes. This study provides sex-specific information on MS activity interventions. Future studies should use improved designs to explore more sociodemographic (e.g., health status, marital status and smoking status) and other dimensional correlates of MS activities among Chinese adults, to provide an evidence base for improved health behavior interventions.

## 1. Background

Globally, it is thought that non-communicable diseases (NCDs), such as cardiovascular diseases, cancers, diabetes, and so on, are mainly responsible for morbidity and morality [1]. For example, over 60% of all death across the world are the result of NCDs [1,2]. In 2016, about 90% of deaths in the U.S. were instigated by NCDs [3]. Physical activity (PA) is a well-recognized preventable factor to reduce NCDs because of the compelling evidence base [4]. The World Health Organization (WHO) [4,5] and some national governments [6] issued PA guidelines which encourage at least 150 min of PA per week for adults. However, data shows that the prevalence of meeting the PA guidelines among adults is about 40% to 60% in the world [7], suggesting these populations are inadequately active for desirable health outcomes [8]. Consequently, promoting sufficient PA has been listed as an essential 21st century public health priority globally [9,10,11].

In addition to recommending overall PA level, the PA guidelines suggest that adults should engage in muscle-strengthening (MS) activities regularly [4,5,6]. MS activities can be defined as any activity causing a muscle contraction against an external resistance (e.g weight training) or the person’s own bodyweight (e.g push-ups), with the expectation of increased strength [12]. They have extra and independent health benefits. From epidemiological surveys and experimental studies, the evidence suggest that regular participation in MS activities may be associated with reduced risk of sarcopenia, osteoporosis, and osteoarthritis, reduced risk of metabolic syndrome and type 2 diabetes [13,14], and lower levels of symptoms of anxiety [14]. Thus, to be specific, the WHO PA guidelines, as well as other national guidelines, recommend that adults aged 18–64 years old should do MS activities at least two days a week [2,4,6].

Relative to the large number of studies on overall PA [7,9,15,16], studies on MS activities among adults remain sparse [17,18]. Over the past several years, increasing numbers of studies have investigated MS activities among different populations. For example, using a nationally representative sample, Bennie et al. [18] reported that 18.6% of adults met the MS activities guidelines. Among US adults, a study including 397,423 samples showed that the proportion of people meeting the MS activities guidelines was 30.2% [19]. Studies have also described the level of MS activities among Scottish [20] and Finnish [21] populations. However, those studies were based on Western populations and there has been less research on Asian populations. In particular, very few studies have examined the prevalence and correlates of MS activities in a large sample of Chinese adults, which inhibits researchers’ understanding of Chinese adults’ MS activities. Indeed, Chinese adults are exposed to health risks owing to insufficient PA and excessive sedentary behavior [22,23]. As an important component of PA, MS activities are significantly associated with improved health. On this point, it is urgent and necessary to investigate the level of MS activities among Chinese adults.

Moreover, researchers have called for effective interventions to improve the population’s MS activities [12,17,20]. Prior to the effective interventions, correlates of MS activities should and must be fully understood. The social ecological model (SEM) [24] is a framework which can be used to explore the correlates of PA, including its subsets (e.g., MS activities). The SEM categorizes correlates of PA into five dimensions, which are the individual, social, environment, policy, and global aspects [15]. The first two dimensions (mainly refers to sociodemographic correlates) have been frequently studied to determine their effects on PA among different populations [15,25]. Recently, several studies have investigated sociodemographic correlates which are associated with MS activities among adults. For example, Freeston et al. [12] indicated that living residence and age were only two factors related to MS activities in the fully adjusted models among Australian adults. These findings were supported by Bennie et al. [17], in a paper published in 2016. Bennie et al. [17] further added that lower education and income were negatively associated with lower frequency of MS activities. In Vezina et al.’s [26] study, sociodemographic factors such as sex, age, education, and race/ethnicity were significantly associated with higher frequency of MS activities. Based on this evidence, researchers can design targeting and effective interventions to. Unfortunately, there is no evidence regarding the correlates of MS activities among Chinese adults, which impedes the capacity to implement MS activity promotion actions. To better understand MS activities among Chinese adults, evidence of correlates of the MS activities is required, based on empirical studies. For this regard, it is recommended that Chinese researchers should explore the correlates of MS activities among inhabitants of China.

In addition to understanding the correlates of MS among Chinese adults, there is also a need to comprehend behavior specificity in the PA research field. It is well-known that overall PA consists of various types, like exercise, chores, and others. In the behavioral research, establishing predictions between characteristics and outcomes is important. However, the predictive capacity differs across the broad range of activities. Hence, finding specific correlates of one form of PA is a priority area for current research [27]. On this point, studying the correlates of MS activities among adults is theoretically meaningful, which should inspire research interest.

To address the aforementioned evidence gaps in the literature, the aims of this study were, therefore, to determine the prevalence of MS activities which follow the guidelines among a sample of Chinese adults in a region of the mainland and to investigate sociodemographic correlates (because of their popularity in the previous literature).

Our reasoned hypotheses, based on previous studies were the following:

1. being male is positively associated with MS activities;
2. age is negatively associated with MS activities;
3. living in rural areas is positively associated with MS activities;
4. higher education levels and income are positively associated with MS activities;
5. full-time job is positively associated with MS activities (this was because having a job is an indicator of people’s socio-economic status (SES); evidence has shown that higher levels of SES are associated with more MS activities, like higher levels of education and increased income, and hence it was expected that full-time job would be positively associated with MS activities in our study);
6. being overweight or obese is negatively associated with MS activities;
7. family composition is significantly associated with MS activities (because there was no evidence regarding family-related indicators and MS activities, according to the SEM models that family’s characteristics are associated with PA, we hypothesized that family composition is associated with MS activities);
8. the relationships between the sociodemographic correlates and level of MS activities differ by sex.

## 2. Methods

### 2.1. Study Design and Participants

This study was a cross-sectional survey which focused on investigating parental influences on children’s active lifestyles, conducted from May to June 2019 in the Hubei Province of China. Within Hubei Province, the commission of education was contacted in each of its thirteen cities. Using convenience sampling, three public schools (one primary school, one middle school, and one high school) were invited from each city to participate in the survey. The administrative support within each commission of education allowed for the selection of 3rd to 12th grade students from 39 public primary, middle, and high schools. In each grade, students of one class were selected randomly. In total, 6583 students (aged 10 to 18 years) were invited to participate in the survey. Data were collected and analyzed anonymously. In response, 5898 students and their parent (either mother or father; response rate = 89.6%) completed the self-reported questionnaire. The questionnaire consisted of 30 questions, such as participants’ sociodemographic information (e.g., sex, age and residence), physical activity and sedentary time (using the International Physical Activity Questionnaire; IPAQ), attitudes towards their children’s exercise and MS activities. Finally, 3073 adult-participants provided valid information for all variables pertaining to this study. The study protocol and procedure were approved by the Institutional Review Board (IRB) of Wuhan University of Technology in March 2019 (WUT No. 2019030016). Participants and their legal guardians provided written consent. To improve the reliability and validity of the survey, the research staff promised that participants’ information will be strictly protected and treated anonymously.

### 2.2. Measurements

#### 2.2.1. MS Activities

Participants were asked whether they had conducted any MS activities in the previous week. An affirmative response warranted further questioning, namely “how many times did you do MS activities last week?” In this context, MS activities were defined as “activities to be done involving major muscle groups [4], like push-up, weightlifting, curl-up or pull-up” to help participants better understand and fill in the questionnaire easily. Response times for participants were 0–7 days. Similar questions had previously been used in studies based on US [19,27] and Australian [12,18] populations, which showed acceptable reliability [28]. In accordance with WHO [4] and consistent with past studies [12,18,19] (two or more days for MS activities as a cut-off), participants were classified as meeting the MS recommendations if they reported having participated in MS activities two or more times per week. For the purpose of statistical analysis, MS activities were summarized as a binary variable (met VS not met).

#### 2.2.2. Sociodemographic Factors

Selected sociodemographic factors were identified based on previous studies [18,19,26]. Participants were required to report their sociodemographic information. This included sex (male or female), age (years as integer), residence (urban or rural), occupational status (employed or unemployed), education level (no college/university or college/university or higher), personal annual income (<9000; 9001–30,000; 30,001–100,000; >100,000) based on previous measurements [29,30,31], and family composition (single children or multiple children), as well as height (cm) and weight (kg), which were used to calculated body mass index (BMI). Participants with a BMI ≥25.0 were viewed as overweight or obese (OW/OB). In the statistical analysis, age was treated as a categorical variable, which was divided into three progressive groups according to participants’ responds (30–40 years; 41–50 years; 51–65 years and above).

### 2.3. Statistical Analysis

Descriptive statistics were used to report the percentage participating in MS activities (met VS not met) and sociodemographic correlates (sex: male or female; age groups: 30–40 years or 41–50 years or 51–65 years and above; residence: urban or rural; occupations status: full job or no job; weight status: normal or overweight/obese; educational level: under college/university, or college/university or higher; family composition: single or multiple children; personal income: <9000 or 9000–30,000 or 30,000–100,000 or >100,000). Pearson’s chi-squared tests were used to detect differences in MS activities among the sociodemographic correlates. A series of multiple logistic regression analyses was conducted to examine the associations of MS activities with sociodemographic characteristics. Adjusted odds ratios (aORs) with corresponding 95% confidence intervals (95% CI) were described. Statistical analyses were performed using IBM SPSS 24.0 software.

## 3. Results

Final data were available for 3073 participants in this study (males accounted for 43.6%). Table 1 shows that 28.5% of the participants in the overall sample met the MS activities guidelines. The Chi-squared test analysis revealed some differences in meeting the MS activities guidelines across different sociodemographic factor levels. In particular, the male participants reported higher levels of meeting the MS activity guidelines compared to their female counterparts (*p* = 0.000). Moreover, participants aged 30–40 years reported the lowest percentage of meeting the MS activities guidelines (26.5%) compared to the 41–50 years and 51 and above years groups (*p* = 0.009). Another significant difference was observed in participants with different family compositions (single children = 26.7% vs. multiple children = 34.1%, *p* = 0.000). Significant differences were not detected among the other sociodemographic groups (*p* > 0.05).

Table 2 summarizes the discrepancies in meeting the MS activities guidelines according to numerous sociodemographic factors, stratified by sex. Among the female participants, there was no significant difference in meeting the MS activities guidelines for various sociodemographic factors (*p* > 0.05). The male participants, however, did show disparities. For instance, those who were OW/OB, or had only one child, reported a higher prevalence of meeting the MS activities guidelines (31.4% and 33.6%, respectively) compared to their female counterparts (both *p* < 0.05).

Results of the multivariate logistic regression are presented in Table 3. In the overall samples, only sex and family composition were significantly associated with meeting the MS activities guidelines. More specifically, male participants were more likely to meet the MS activities guidelines than females (aOR = 1.98, 95%CI: 1.67–2.34). Adults with two or more children had a higher chance of meeting the MS activities guidelines (aOR = 1.35, 95%CI: 1.12–1.64). In the male samples, adults who were not overweight or obese were more likely to meet the MS activities guidelines (aOR = 1.36, 95%CI: 1.08–1.78) compared to their counterparts. Such a significant relationship was also observed between family compositions and meeting the MS activities guidelines in the male samples (aOR = 1.58, 95%CI: 1.21–2.05). It is noteworthy that in the female samples, there was no evidence of significant relationships between sociodemographic correlates and meeting the MS activities guidelines.

## 4. Discussion

To our knowledge, this study is the first to examine the level of MS activity among adults in mainland China. An important finding of this study was that a minority of Chinese adults in Hubei Province (28.5%) met the MS activities guidelines. Another key finding was that in the fully adjusted model, the sociodemographic factors of sex (male) and family composition (multiple children) were positively associated with MS activities among Chinese adults in mainland China. Further, the correlates of MS activities differed between the specific sexes. The findings can be applied practically in two ways—to inform policy and intervention designers. For the former, it is a crucial to raise awareness of the importance of MS activities—particularly among Chinese adults in Hubei Province—and to promote MS activities efficiently. For the latter, PA behavioral research is needed to establish more of the sociodemographic correlates of MS activities, which will allow for a better understanding of this form of PA among adults.

The overall prevalence of Chinese adults in Hubei Province who reported having MS activities in the current study (28.5%) was generally higher than those reported in previous studies of Australian populations [12,18] but was still lower than some reports in the US (30.2%) [19]. This may be attributed to differences in sample characteristics. For example, the study in the US [19] included samples aged 18–30 years old, who can be regarded as younger people. It has previously been documented that younger people had higher levels of MS activities [12], which may make their results higher. Another possible explanation for inconsistent results across the literature is measurement differences. In Vezina et al.’s study [26], the authors employed an item to collect participants’ information on MS activities over the past month. This measurement might result in a lower estimation of MS activities, owing to limited cognitive capability. Such a discrepancy stresses the necessity for a standardized measurement of this PA behavior. However, despite higher reports of MS activities than in some other surveys, it should be acknowledged that a lower proportion (28.5%) of Chinese adults in the Hubei Province were performing recommended MS activities (at least two days a week). Owing to the various health benefits of MS activities to adults [13,14,32,33,34], improving their MS activity participation is a pressing matter.

Among the total samples, males were more likely than females to report the recommended level of MS activity (two or more days a week). This finding is consistent with previously published studies based on regional or national samples [12,17,18,19]. One possible explanation is that males are perhaps more conscious of bodily stature, which evokes interest in MS activities. Additionally, owing to traditional Chinese social norms and cultural practices, females are not encouraged to participate in muscle-related activities, because of aesthetic values. However, considering the health benefits of MS activities [33,34], targeting the female population as an interventional priority is necessary. The current study found that family composition was a significant correlate of MS activities among Chinese adults (as well as being male). This significant finding was not previously established. However, limitations in evidence mean that there is little support for the finding. Hence, more evidence is needed to confirm this result and explore its underlying mechanism.

In contrast to prior research [2,3,4], this study found that age, residence, weight status, educational level, occupation, and income were not significant correlates of MS activities among the adult sample. Concerning age, studies based on Western populations found younger adults were more likely to participate in MS activities compared to older adults [17,18,19,26]. This study did not confirm this, partially because of the different cut-offs among the age group categories of the samples in this study. Residence was associated with MS activities among Australian adults, which is not in line with this study [11]. A possible reason for this might be environmental differences—it may be easier to access more space for MS activities in rural areas than urban areas in Western countries [11]. However, such a difference may not be replicated in the Hubei Province, which displays no difference in MS activities between different residential areas. Weight status was identified as a significant correlate of MS activities in US adults [2], but not among Australian adults [3]. This study only partially supports some of the previous reports [3]. Concerning weight status, the current findings suggest that the impact of this correlate on MS activities among adults requires further examination. Educational level, occupation, and income can be considered socio-economic indicators, which represent knowledge, skills, and materials. A study by Freeston et al. [12] indicated that the aforementioned factors are not associated with MS activities among adults according to their fully adjusted analysis—this supports the findings of this project. Generally, adults with developed socio-economic indicators show better health behaviors, including greater engagement in MS activities, due to improved health-related knowledge and skills. However, in our study samples, participants with developed socio-economic indicators did not demonstrate higher levels of MS activity. A plausible interpretation is that the participants may lack relevant knowledge and skills for engagement in MS activities. Overall, the inconsistencies between this study and others need to be addressed, a requirement for future research in this area.

Interestingly, the overweight/obese factor was a negative correlate of MS activities among male adults. As mentioned above, males were more likely to pay attention to their figure and so focus more on MS activities to maintain an ideal body shape or size. As a result, males spend more cumulative time on MS activities. It is therefore possible that males with normal weight status are more likely to report higher levels of MS activity.

In this study, there were no significant relationships between sociodemographic correlates and MS activities among female adults. This finding implies that there might be other sociodemographic factors affecting MS activities among females in the Hubei Province. Thus, it would be beneficial to expand on this study by assessing more specific sociodemographic factors that impact engagement in MS activities, specifically amongst female adults. These additional factors can be considered for designing interventions involving MS activities for the female population.

### Limitations of This Study

This study presented some limitations. First, the cross-sectional design could not determine causality. Thus, the real causal relationship of some sociodemographic factors with MS activities is unknown. Second, the self-reported measure was used to obtain information on MS activities, which enabled recall bias [35], like for income, body weight and height. Third, our data only represents the regional situation, which may limit the generalization of our research findings to the wider society. Thus, application of the research findings to the wider population would need to be done with caution. Fourth, owing to measurement limitations, we were not able to capture more information on MS activities like duration, type, or location. This resulted in uncertainties about the profiles of MS activities engagements among adults. Future studies should determine more correlates of MS activities among Chinese adults, in order to expand on the current research.

## 5. Conclusions

Less than 30% of Chinese adults in the Hubei Province met the recommended levels of MS activity. These activities were associated with sex and family composition. The relationships between the sociodemographic correlates and level of MS activity also differed by sex. This study may advance understanding of MS activities among adults. For public health benefits, large efforts should be made to increase participation in MS activities among adults. Future public MS activities interventions should prioritize female adults, those who have multiple children, and overweight/obese male adults, all of whom demonstrated low MS activity engagement.

## Figures and Tables

**Table 1:** Prevalence of meeting the muscle-strengthening (MS) activities guidelines by sociodemographic factors.

Variables	Met Muscle-Strengthening Activities Guidelines	
	*n*	%
Overall	875	28.5
**Sex**		
Male	487	36.3
Female	388	22.4
*p*	0.000	
**Age**		
30–40	421	26.5
41–50	408	30.0
51–65	46	37.4
*p*	0.009	
**Residence**		
Urban	685	27.9
Rural	190	30.7
*p*	0.171	
**Weight Status**		
Overweight	686	28.3
Obesity	189	29.1
*p*	0.680	
**Family Composition**		
Single children	628	26.7
Multiple children	247	34.1
*p*	0.000	
**Educational level**		
Under college or university	628	28.8
College or university or higher	247	27.6
*p*	0.475	
**Occupation**		
Full job	844	28.6
No job	31	25.8
*p*	0.513	
**Personal Income (annually)**		
<9000	137	29.0
9000–30,000	257	29.9
30,000–100,000	341	26.8
>100,000	140	29.9
*p*	0.354	

**Table 2:** Prevalence of meeting the muscle-strengthening (MS) activities guidelines by sociodemographic factors and sex.

Variables	Met Muscle-Strengthening Activity Guidelines			
	Male		Female	
	*n*	%	*n*	%
**Age**				
30–40	179	35.2	242	22.4
41–50	269	36.6	139	22.2
51–65	39	40.2	7	26.9
*p*	0.622		0.852	
**Residence**				
Urban	373	35.8	312	22.1
Rural	114	38.3	76	23.7
*p*	0.430		0.544	
**Weight Status**				
Normal	356	38.5	330	22.0
Overweight/obesity	131	31.4	58	25.0
*p*	0.012		0.308	
**Family Composition**				
Single children	334	33.6	294	21.7
Multiple children	153	44.2	94	24.9
*p*	0.000		0.193	
**Educational level**				
Under college or university	346	37.2	282	22.6
College or university or higher	141	34.4	106	21.8
*p*	0.330		0.712	
**Occupation**				
Full job	478	36.2	366	22.4
No job	9	47.4	22	21.8
*p*	0.313		0.878	
**Personal Income (annually)**				
<9000	77	34.8	60	23.9
9000–30,000	139	37.8	118	24
30,000–100,000	197	34.9	144	20.3
>100,000	74	39.6	66	23.5
*p*	0.590		0.384	

**Table 3:** Associations of sociodemographic correlates with meeting the MS activities guidelines.

Sociodemographic	Met Muscle-Strengthening Activities Guidelines								
	Overall			Male			Female		
	Adjusted OR	95%CI		Adjusted OR	95%CI		Adjusted OR	95%CI	
*Sex*									
Male	1.98	1.67	2.34	/			/		
Female	ref								
*Age*									
30–40	0.80	0.54	1.19	0.78	0.49	1.23	0.76	0.31	1.87
41–50	0.85	0.57	1.26	0.87	0.56	1.35	0.76	0.31	1.88
51–65	ref			ref			ref		
*Residence*									
Urban	0.96	0.78	1.18	0.97	0.73	1.30	0.95	0.70	1.29
Rural	ref			ref			ref		
*Weight Status*									
Normal	1.17	0.96	1.43	1.39	1.08	1.78	0.87	0.63	1.20
Overweight/obesity	ref			ref			ref		
*Family Composition*									
Single children	ref			ref			ref		
Multiple children	1.35	1.12	1.64	1.58	1.21	2.05	1.15	0.87	1.51
*Educational level*									
Under college or university	ref			ref			ref		
College or university or higher	0.93	0.77	1.12	0.88	0.68	1.15	0.98	0.75	1.30
*Occupation*									
Full job	1.02	0.66	1.57	0.67	0.27	1.70	1.11	0.67	1.83
No job	ref			ref			ref		
*Personal Income (annually)*									
<9000	ref			ref			ref		
9000–30,000	1.11	0.86	1.42	1.18	0.83	1.68	1.01	0.71	1.45
30,000–100,000	0.98	0.77	1.25	1.16	0.83	1.63	0.84	0.59	1.19
>100,000	1.19	0.89	1.60	1.41	0.93	2.15	1.01	0.67	1.53

OR: odds ratio; CI: confidence interval; ref: reference group in the logistic models.
